# Alpha-Glucosidase Inhibitors Alter Gut Microbiota and Ameliorate Collagen-Induced Arthritis

**DOI:** 10.3389/fphar.2019.01684

**Published:** 2020-02-04

**Authors:** Lingshu Zhang, Pingfang Song, Xiaowei Zhang, Christina Metea, Matthew Schleisman, Lisa Karstens, Eric Leung, Jun Zhang, Qiang Xu, Yi Liu, Mark Asquith, Cong-Qiu Chu

**Affiliations:** ^1^ Division of Arthritis and Rheumatic Diseases, Oregon Health & Science University, Portland, OR, United States; ^2^ Department of Rheumatology, West China Hospital, Sichuan University, Chengdu, China; ^3^ Section of Rheumatology, VA Portland Health Care System, Portland, OR, United States; ^4^ Casey Eye Institute, Oregon Health & Science University, Portland, OR, United States; ^5^ Department of Medical Informatics and Clinical Epidemiology, Oregon Health & Science University, Portland, OR, United States; ^6^ Department of Surgical Oncology, University of Texas MD Anderson Cancer Center, Houston, TX, United States; ^7^ Department of Rheumatology, The First Hospital, Guangzhou University of Chinese Medicine, Guangzhou, China

**Keywords:** acarbose, arthritis (including rheumatoid arthritis), microbiome, Th17, Treg cells

## Abstract

Acarose is an anti-diabetic drug and exhibits anti-arthritic effects. We hypothesized that acarbose influences the gut microbiota to affect the course of arthritis and tested this hypothesis in a collagen-induced arthritis (CIA) murine model. Acarbose in drinking water was administered *via* gastric gavage started prior to or at the time of CIA induction. Gut microbiota were evaluated with 16S rRNA gene sequencing from fecal pellets collected prior to arthritis induction, during onset of arthritis, and after treatment. Immune response was evaluated by measuring changes in T helper-17 (Th17) and T regulatory (Treg) cells in the spleen and intestine, as well as serum cytokine levels. Before induction of CIA, acarbose significantly reduced the incidence of arthritis and attenuated clinical severity of arthritis. The frequency of Th17 cells was significantly decreased in the intestinal lamina propria in acarbose treated mice. Mice that were treated with acarbose showed significantly increased CD4^+^CD25^+^Foxp3^+^ Treg cells with elevation of Helios and CCR6. A remarkable alteration in microbial community was observed in acarbose treated mice. Bacterial diversity and richness in mice with arthritis were significantly lower than those in acarbose treated groups. The frequency of *Firmicutes* was significantly reduced after arthritis onset but was restored after treatment with acarbose. The frequency of *Lactobacillus*, *Anaeroplasma*, *Adlercreutzia*, *RF39* and *Corynebacterium* was significantly higher in control groups than in acarbose treated, while *Oscillospira*, *Desulfovibrio* and *Ruminococcus* enriched in acarbose treated group. Miglitol, another α-glucosidase inhibitor showed a similar but less potent anti-arthritic effect to that of acarbose. These data demonstrate that acarbose alleviated CIA through regulation of Th17/Treg cells in the intestinal mucosal immunity, which may have resulted from the impact of acarbose on gut microbial community. Inexpensive antidiabetic drugs with an excellent safety profile are potentially useful for managing rheumatoid arthritis.

## Introduction

Acarbose is the prototype of alpha-glucosidase inhibitors and a widely used anti-diabetic drug. Acarbose is a pseudo-carbohydrate that competitively inhibits α-glucosidase, which is located in the brush border of the small intestine, leading to the delay of intestinal absorption of carbohydrates and therefore slows down the absorption of sugars (Bischoff, 1994), and reduced postprandial increases in blood glucose. Acarbose is primarily prescribed to patients with type 2 diabetes as monotherapy or as add-on to other antidiabetic drug treatment (Joshi et al., 2015), especially in those with poorly controlled blood glucose by other antidiabetic drugs. Additional benefits have been noticed in diabetic patients treated with acarbose such as reduced risk for myocardial infarction and improved lipid profile and reduced levels of inflammatory cytokines in peripheral blood (Hanefeld and Schaper, 2008; Joshi et al., 2015; Su et al., 2015).

Recently, it has been observed that diabetic patients treated with acarbose have a decreased risk for developing rheumatoid arthritis (RA) (Chen et al., 2015) suggesting an anti-inflammatory effect by acarbose. The anti-inflammatory effect of acarbose has been experimentally proven in two mouse models of inflammation, namely collagen-induced arthritis (CIA) and a psoriasis model (Chen et al., 2015; Chen et al., 2016). In the two models of inflammatory diseases, daily administration of acarbose attenuated the diseases severity.

RA is a chronic autoimmune disease involving inflammation of the synovial joints that leads to cartilage degradation, bone erosion, and joint destruction. The cause of RA remains obscure, but interplay of genetic and environmental risk factors are thought to be responsible. Recently, dysbiosis of the gut microbiota has been considered as a contributor to RA etiopathogenesis (Scher and Abramson, 2011; Phillips, 2015; Rogers, 2015). Interestingly, the microbiota dysbiosis in RA can be partially normalized by disease-modifying anti-rheumatic drugs (DMARDs) in those RA patients achieved remission of low disease activity (Zhang et al., 2015) indicating that gut microflora biosis is associated with RA disease status. Patients with diabetes treated with acarbose have increased gut *Biffidobacterium longum* and *Enterococcus faecalis* (Su et al., 2015). Moreover, mice fed with acarbose have increased lifespan (Harrison et al., 2014; Harrison et al., 2019). The enhanced longevity in acarbose treated mice is associated with changes in the gut microbiome (Smith et al., 2019). Given the linkage of decreased risk of developing RA in diabetic patients treated with acarbose, anti-inflammatory effect of acarbose in CIA, alteration of gut microbiome in mice treated with food supplement with acarbose, we hypothesized that acarbose exerts anti-inflammatory effects *via* alteration of the gut microbiota, and tested this idea in CIA by analyzing changes of bacterial composition before and after treatment with acarbose. Since CIA is known to be T helper 17 (Th17) cell dependent and displays T regulatory (Treg) cell functional defect (Chu et al., 2007; Morita et al., 2011), we focused on the immune changes of Th17 and Treg cells in the intestine and correlating them with changes of gut microbiota after acarbose treatment.

## Materials and Methods

### Mice and Induction of CIA and Clinical Assessment of Arthritis

Male DBA/1 mice (8–10 weeks old) were purchased from the Jackson Laboratory (Bar Harbor, ME). All mice were maintained in a specific, pathogen-free facility and housed in microisolator cages containing sterilized food, bedding and water. All animal experiments were performed under protocols approved by Institutional Animal Care and Use Committee of VA Portland Health Care System. Arthritis was induced by immunization with intradermal injection of 100 μg of chicken type II collagen (CII) (Chondrex, Inc., Seattle) emulsified 1:1 with complete Freund’s adjuvant (CFA) and boosted on day 35 with an intraperitoneal injection of 25 μg of bacterial lipopolysaccharide (LPS). All mice were examined daily for the initial visual appearance of arthritis. Severity of arthritis was classified using a scoring system as described (Chu et al., 2007). Score 0: No evidence of erythema and swelling; Score 1: Erythema and mild swelling confined to the tarsals or ankle joint; Score 2: Erythema and mild swelling extending from the ankle to the tarsals; Score 3: Erythema and moderate swelling extending from the ankle to metatarsal joints; Score 4: Erythema and severe swelling encompassing the ankle, foot and digits, or ankylosis of the limb. The maximum score of each mouse is 16.

### Treatment Protocols

**Figure 1:**
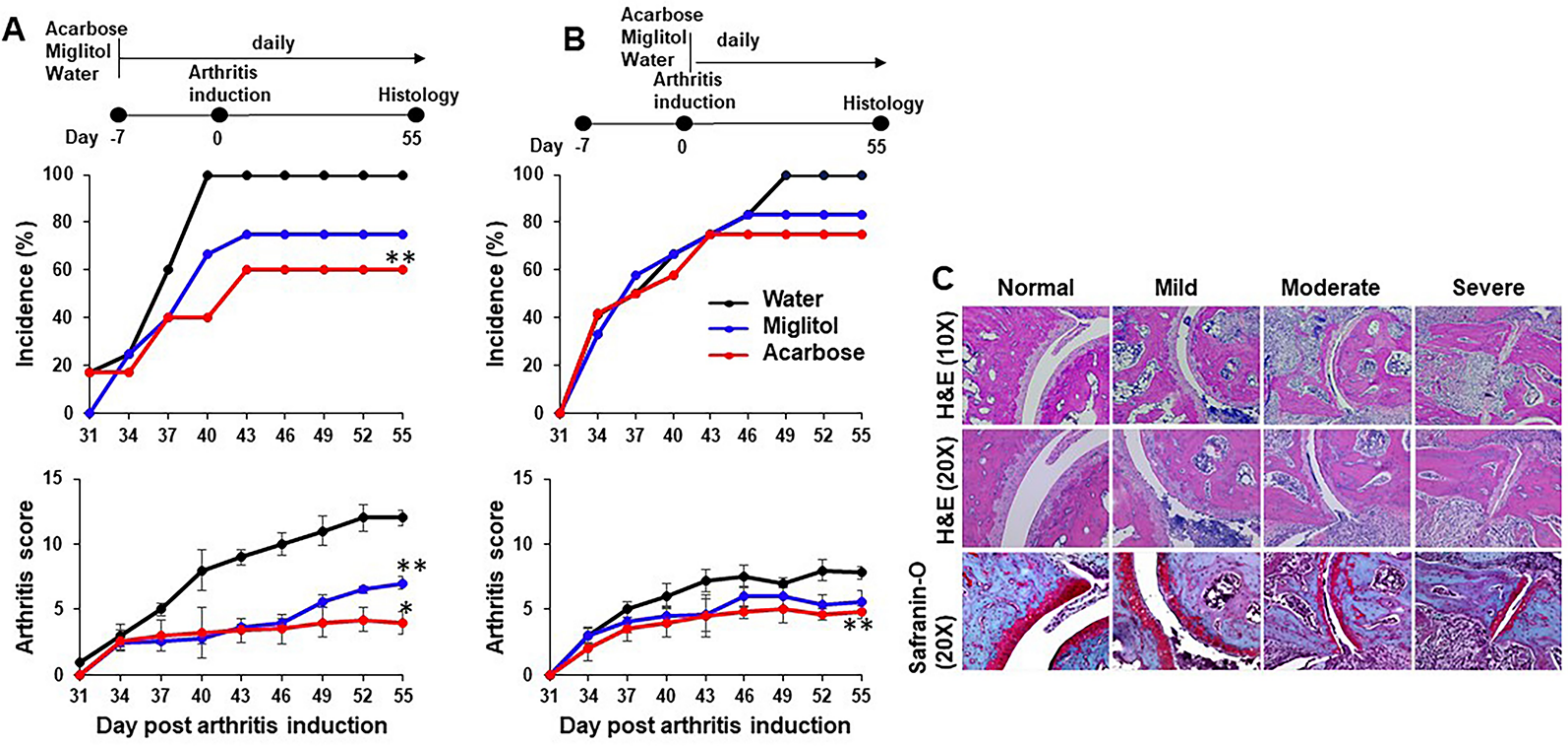
Acarbose protected against collagen-induced arthritis. DBA/1 mice were treated with acarbose or miglitol or water either 7 days prior to **(A)**, Before Induction or at the time of arthritis induction (day 0) **(B)**, At Induction. Incidence and severity of arthritis were evaluated until day 55 post arthritis induction. **(C)** Joints of mice were harvested and stained with hematoxylin and eosin (H&E) and safranin-O. Representative photographs of histopathology showing synovitis, pannus formation, and marginal erosion of the joint and articular architectural changes in normal joint and mild, moderate and severe arthritis (pooled data of two experiments, n = 10–15 mice in each group). *p < 0.01; **p < 0.05.

To investigate whether acarbose prevents arthritis and suppresses joint destruction *in vivo*, we developed two experimental protocols based on published data (Chen et al., 2015; Chen et al., 2016). In the first protocol (**Figure 1A**), mice were gavaged with 300 μl of acarbose 500 mg/kg, miglitol 500 mg/kg or drinking water daily starting at 7 days prior to induction of arthritis (before induction). In another protocol (**Figure 1B**), mice were gavaged with 300 μl of acarbose 500 mg/kg, miglitol 500 mg/kg or drinking water every day starting on the day of arthritis induction (at induction). All mice were euthanized on day 55 after arthritis induction.

### Cell Isolation and Purification of Spleen and Intestinal Lamina Propria T Cells

Spleen was removed and cells were isolated *via* single cell suspension by mechanical disruption through a 100 μm cell strainer. Lysis of erythrocytes was performed for spleens. To isolate cecum and colon intestinal lamina propria (LP), cecum and colons were removed, opened longitudinally, contents were flushed with ice-cold PBS containing 0.1% bovine serum albumin (BSA). Intestines were cut into 3 mm in length and incubated for 30 min at 37°C with RPMI 1640 containing 3% fetal bovine serum (FCS), 5 mM EDTA, 10 mM HEPES, 100 U/ml penicillin, and 100 μg/ml streptomycin (Gibco, USA) with and 1 mM dithiothreitol (DTT, Fisher Scientific, Pittsburgh) with gentle shaking at 37°C for 30 min to remove the epithelium and intraepithelial lymphocytes. Tissues were washed with PBS containing 0.1% BSA twice, then followed by incubation with 0.2 mg/ml collagenase II (Sigma) and 0.1 mg/ml DNase I (Roche) with stirring at 37°C for 45 min. Cells were then filtered through a 40 μm cell strainer and harvested at the interface of a 40%/70% Percoll (GE Healthcare) gradient, then washed in PBS containing 0.1% BSA for assays.

### Flow Cytometry

Intracellular staining was performed as follows. Cells were stimulated with phorbol myristate acetate (50 ng/ml, Calbiochem) plus ionomycin (500 ng/ml, Merck & Company) for 4 h, with Golgi Plug (BD Biosciences). After cell surface staining with antibodies to CD4, CD25 and CCR6 (BioLegend), cells were fixed and permeabilized Fix/Perm buffer (eBioscience) overnight at 4°C. Antibodies to Helios, IL-17A and Foxp3 (eBioscience) were added and incubated for 30 min at 4°C. Cells were acquired with a BD LSRII and data were analyzed using FlowJo (V10, USA) software.

### Histopathology of Arthritis

Mouse joint tissues were removed, fixed in 10% formalin, decalcified, embedded in paraffin and sectioned at 6 μm. Sections were stained with hematoxylin and eosin, Safranin-O and toluidine blue to detect synovitis, pannus formation, and marginal erosion of the joint and articular architectural changes.

#### Anti-CII Antibody Quantification

Mouse serum samples were collected at the end of the experiment. Antibodies of different isotypes to chicken and murine CII (Chondrex, Seattle, WA) were determined by enzyme linked immunosorbent assay (ELISA), as previously described (Chu et al., 2003). Microtitre plates were coated with 5 μg/well of native chicken or murine CII in potassium phosphate coating buffer (pH 7.4) overnight at 4°C, followed by blocking with an overnight incubation at 4°C with blocking buffer (2% BSA in PBS). Serum samples diluted at 1:4,000 in PBS containing 0.05% Tween 20 and 0.5% BSA were incubated 1 h at room temperature. Goat anti-mouse IgG, IgG1, IgG2a and IgG2b conjugated to alkaline phosphatase (Jackson ImmunoResearch Laboratorie) diluted at 1:5,000 were incubated for 30 min at room temperature to detect anti-CII or anti-mouse antibody isotypes and IgG subclasses. The optical density at 450 nm was read with a Molecular Devices plate reader (Molecular Devices, Menlo Park, CA). Antibody levels were expressed as arbitrary units with reference to a standard serum.

#### Multiplex Cytokine Assay

Serum levels of various cytokines (IL-2, IL-4, IL-5, IL-6, IL-9, IL-10, IL-13, IL-17A, IL-17F, IL-21, IL-22, IFN-γ, and tumor necrosis factor (TNF)-α) were determined using the LEGENDplex™ Mouse Th Cytokine Panel (13-plex) array (Biolegend) according to the manufacturer’s protocol. The data were collected on a LSR II flow cytometer and analyzed using the LEGENDplex™ software version 7.0 (Biolegend).

#### Fecal Pellet Collection

Fecal pellets for microbial community analysis were collected before arthritis induction (day −7), at arthritis induction (day 0), onset of arthritis (day 37), and at the termination of the experiment (day 55). Fecal samples and cecal contents from mice housed under specific pathogen-free conditions were harvested under sterile conditions in a 1.5 ml Biopure tube (Eppendorf) and immediately snap frozen and stored at −80°C until processing for DNA isolation.

#### 16S rRNA Gene Sequencing

DNA was isolated from fecal pellets and cecal contents using PowerSoil DNA Isolation Kit (MoBio Laboratories Inc., Carlsbad, CA) according to the manufacturer’s instructions. DNA concentration was confirmed by Nanodrop spectrophotometer prior to normalization to ~20 ng/µl. Quantitative real-time PCR (qRT-PCR) amplification was performed using a set of primers targeting the V4 hypervariable regions of the 16S rRNA gene using the 515F–806R primers and sequenced on the Illumina MiSeq platform (Caporaso et al., 2012). The primers used were as follows: FWD : GTGCCAGCMGCCGCGGTAA; REV : GGACTACHVGGGTWTCTAAT (original, rev-barcoded: 515F-806R). Each PCR amplification reaction contained 1 μl of DNA extract, 0.2 mM dNTPs, 0.2 mM forward primer, 1 μl reverse primers, 48 μl of master mix containing 1× colorless reaction buffer, 1.5 mM MgCl_2_ and 1.25 U of polymerase enzyme. The reaction volumes were placed in a thermocycler and run through the following conditions: 94°C for 3 min (initial denaturation), followed by 35 cycles of 94°C for 45 s (denaturation), 55°C for 40 s (annealing) and 72°C for 1.5 min (extension) and ending with a last step of 72°C for 10 min.

The final steps of library preparation were performed with the Illumina Miseq V2 kit. The DNA library was diluted to 4 nM and denatured with NaOH for 5 min. The library was then diluted to a final concentration of 6.6 pM in hybridization buffer HT1 (Illumina), spiked with 5% PhiX control heated to 96°C for 2 min prior to incubation for 5 min on ice and then loaded onto the MiSeq cartridge. A 500 cycle MiSeq Reagent Kit v2 was used and run on the Illumina Miseq instrument.

#### 16S rRNA Gene Sequencing Analysis and Bioinformatics

Primers and sequence adapters were removed with the Illumina MiSeq Reporter (version 2.5) and further processed using the Quantitative Insights Into Microbial Ecology (QIIME) pipeline version 1.9.0 (Caporaso et al., 2010), the Vegan package in R (Dixon, 2003) and Linear discriminant analysis (LDA) effect size (LEfSe) (Segata et al., 2011). To adjust for multiple comparisons the Benjamini–Hochberg method was used (Benjamini and Hochberg, 1995). Taxonomy was assigned with the BLAST consensus method of QIIME, using the most recent version of the GreenGenes database (v.13.8) (DeSantis et al., 2006). Chimeric sequences were detected and removed using the blast_fragments approach implemented in identify_chimeric_seqs.py. Operational taxonomic units (OTU) were picked using UCLUST at 97% sequence identity. Rarefaction was performed to subsample each sample to 17,000 reads prior to comparing the abundance of OTU across samples. We assessed α-diversity with observed OTU count and the Chao1 Estimator for microbial richness. Between group β-diversity was evaluated with unweighted UniFrac distances and principal coordinate analysis in QIIME and statistically evaluated using PERMANOVA. LEfSe analysis was implemented in Galaxy (version1.0) and performed using standard parameters (p < 0.05 and LDA score 2.0).

#### Statistical Analysis

Nonparametric data were compared using Mann–Whitney U test, and parametric data were compared using Student’s t-test. All statistical analyses were calculated in Prism (GraphPad Prism 5). Differences were considered to be statistically significant when P < 0.05.

## Results

### Acarbose Suppressed CIA

Acarbose dissolved in drinking water was administered *via* gastric gavage at 500 mg/kg daily. This dose was chosen based on previously published protocol in which 500 mg/kg was effective while 100 mg/kg did not have effect on arthritis or psoriasis models (Chen et al., 2015; Chen et al., 2016). Miglitol was included as a control drug to acarbose. Miglitol is a second generation of α-glucosidase inhibitor (Sels et al., 1999). While greater than 98% of orally dosed acarbose is not absorbed (Dash et al., 2018), miglitol is completely absorbed by the small intestine (Sels et al., 1999; Dash et al., 2018) and therefore, an appropriate control for gut activity of acarbose. Oral administration of acarbose significantly suppressed CIA when given prior to or at the time of arthritis induction and reduced incidence of CIA when given prior to induction of arthritis (**Figure 1**). The arthritis inhibition effect of acarbose, however, is less potent if given at the time of arthritis induction. The arthritis inhibition effect was demonstrated by histological analysis. In the group of animals treated by acarbose 7 days prior to arthritis induction, 37% of arthritic joints were identified as mild, whereas only 14% of arthritic joints were mild in the drinking water treated group (**Table 1**). In contrast, in the animals treated at induction, 52% of arthritic joints were identified as severe with joint structure destruction in drinking water treated animals, compared with only 16% of the joints belonged to the severe category in acarbose group (**Figure 1** and **Table 1**). Miglitol also had arthritis inhibiting effects but much less potent than acarbose (**Figure 1** and **Table 1**).

**Table 1:** Histopathological severity of arthritis^1^.

Treatment	Number of joints assessed	Severity (%)		
		Mild	Moderate	Severe
Water	35	5 (14)	12 (34)	18 (52)
Miglitol	36	11 (31)#	16 (44)#	9 (25)**
Acarbose	38	14 (37)***	18 (47)#	6 (16)*

*P = 0.001 (Acarbose vs control).

**p = 0.02 (Miglitol vs control).

***p = 0.03 (Acarbose vs control).

#Not statistically significant.

^1^The severity of arthritis in each joint was classified as mild, moderate or severe based on the following criteria: mild, minimal synovitis, cartilage loss and bone erosions limited to discrete foci; moderate, synovitis and erosions present but normal joint architecture intact; severe, synovitis, extensive erosions, and joint architecture disrupted (as shown in **Figure 1C**, (Williams et al., 1992; Chu et al., 2003).

### Modulation of Immune Response by Acarbose

**Figure 2:**
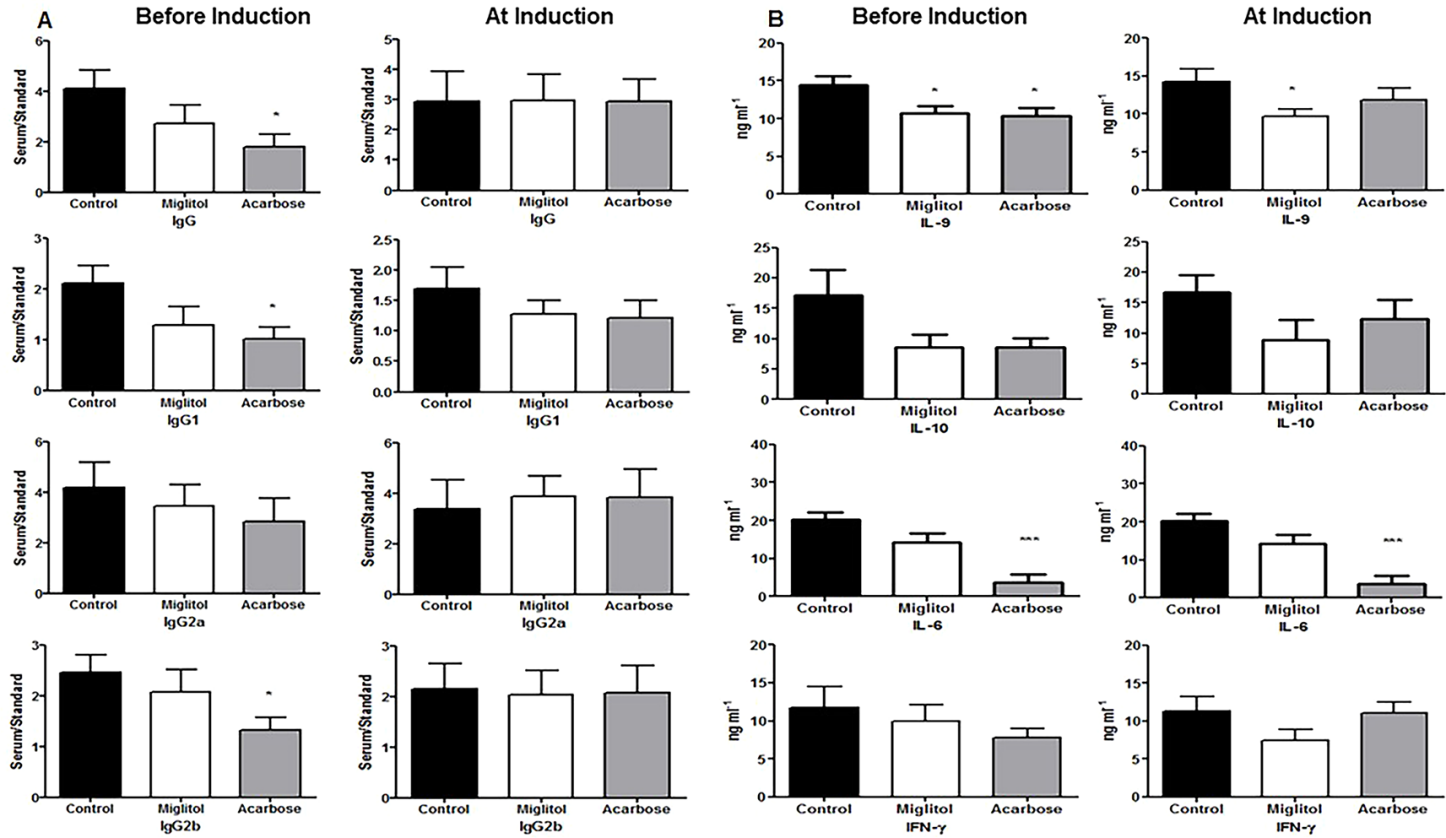
Anti-collagen type II autoantibody and cytokine production. Mice were treated with acarbose, miglitol or water as in **Figure 1** (Before Induction or At Induction). Serum levels of IgG, IgG1, IgG2a and IgG2b anti-collagen type II were determined by specific enzyme-linked immunosorbent assay **(A)** and concentrations of cytokines were determined by the LEGENDplex mouse Th cytokine panel (13-plex) array **(B)**. Data were expressed as mean ± SEM. These results consist of the average of two independent experiments, five mice per group. *p < 0.05, ***p < 0.01, vs control group.

We next sought to identify if α-glucosidase inhibitors modulates the antigen-specific systemic immune response, thereby resulting in protection against CIA. Immunized DBA/1 mice are known to cause elevation of anti-CII antibodies and these antibodies are pathogenic. We measured CII-specific anti-chicken CII and murine CII autoantibody production in serum. Mice treated with acarbose started before arthritis induction showed a significant reduction in levels of anti-chicken CII total IgG, IgG1 and IgG2b antibodies compared to control mice (**Figure 2**). No significant changes were observed in the group of mice treated starting at arthritis induction (**Figure 2**). We did not find any difference between groups in CII type II anti-murine autoantibodies. We measured levels of pro- and anti-inflammatory cytokines. Mice treated with acarbose significantly reduced IL-9 (*P <* 0.05) and IL-6 (*P <* 0.001) while miglitol reduced IL-9 (*P <* 0.05) compared with mice gavaged with water alone in the protocol of treatment starting before arthritis induction. Mice treated with acarbose also showed a significantly decreased level of IL-6 in mice treated at arthritis induction (*P < *0.05). None of the α-glucosidase inhibitors treated mice in both protocols showed any significant changes in cytokines levels of IL-10 and interferon (IFN)-γ compared with water-treated controls (**Figure 2**). For the remaining analyzed serum factors related to T-cell activation status (IL-4, IL-5,IL-13, IL-17A, IL-17F, IL-21, IL-22, IL-23 and TNF), no obvious differences were detected or a comparative analysis was not feasible because of serum levels being predominantly or completely below the detection limit of the applied assays in either mouse group (data not shown).

### Modulation of α-Glucosidase Inhibitor Treatment in Th17 and T Regulatory (Treg) Cells

**Figure 3:**
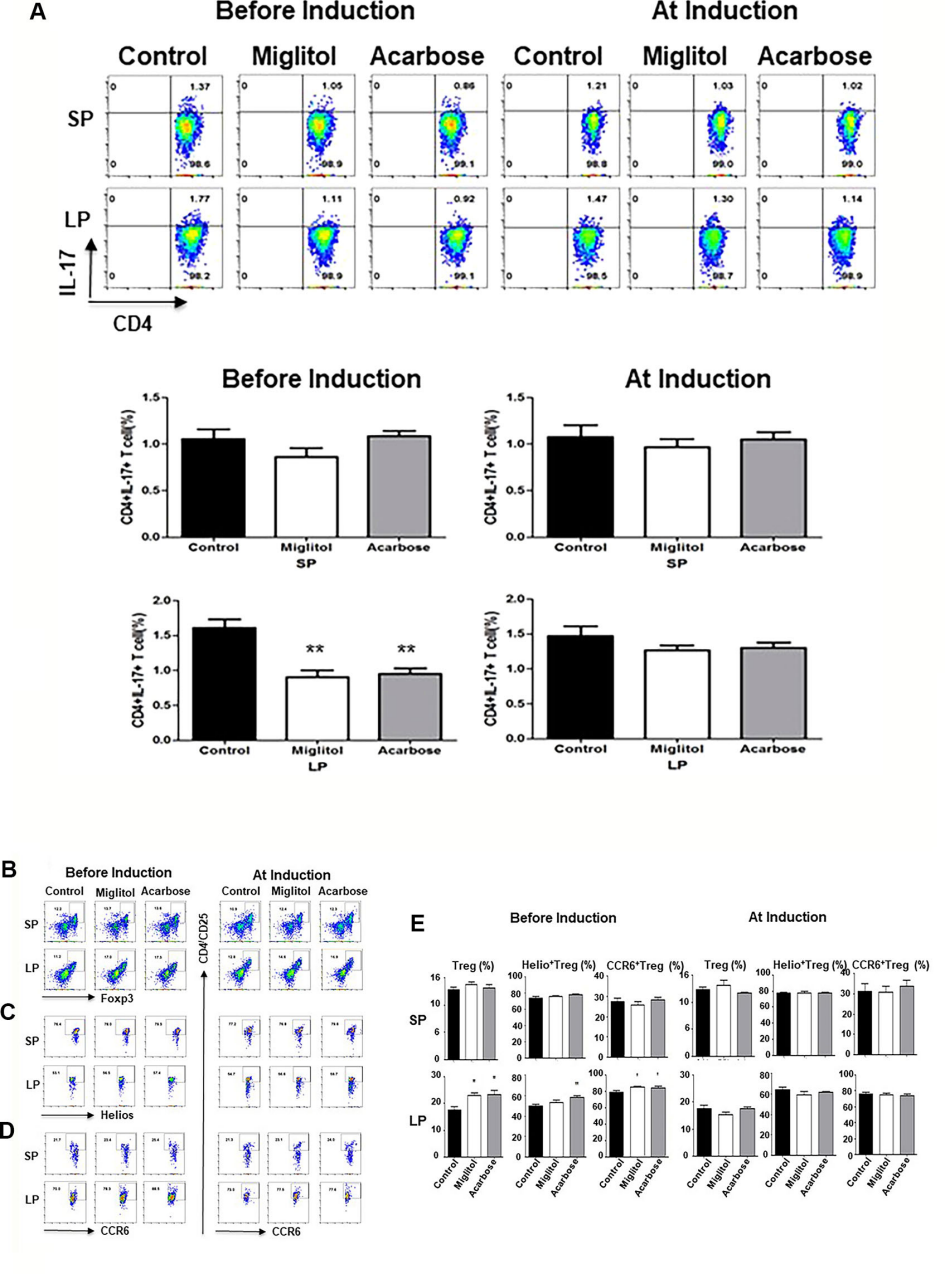
α-glucosidase inhibitor treatment on the mouse spleen and intestinal immune systems. Mice were treated with acarbose, miglitol or water as in **Figure 1** (Befosre Induction or At Induction). The number of Th17 and Treg cells in mouse spleen and gut lamina propria was analyzed using flow cytometry. **(A)** Th17 cells: Lymphocytes were stimulated and stained with CD4 and IL-17A antibodies. Each dot plot shows data from one representative mouse of each group. The number in the right upper quadrant indicates the percentage of CD4+IL-17+ lymphocytes. Bar graphs represent the data expressed as mean ± SEM. **(B**–**E)** Treg cells: Percentage of Treg cells (CD4^+^CD25^+^Foxp3^+^) **(B)**, Helio^+^ Treg cells (CD4^+^CD25^+^Foxp3^+^Helios^+^) **(C)** and CCR6^+^ Treg cells (CD4^+^CD25^+^Foxp3^+^CCR6^+^) **(D)** was quantified by flow cytometry. Each dot plot shows data from one representative mouse of each group. **(E)** Bar graphs represent the data of total Treg, Helio^+^ Treg and CCR6^+^ Treg cells expressed as mean ± SEM. These results were average of two independent experiments, 7-10 mice per group. *p < 0.05, **p < 0.01, vs control group.

To confirm that the treatment with α-glucosidase inhibitors modulate innate and intestinal immune system, we compared spleen and large intestine LP lymphocytes by flow cytometry. We focused on Th17 and Treg cells since Th17 and Treg imbalance is involved in the pathogenesis of CD4^+^ T cells in DBA/1 mice during CIA (Chu et al., 2007; Morita et al., 2011). There was no difference of Th17 cells was detected in the spleen in either treatment protocols, or in gut LP in mice treated at arthritis induction groups. On the other hand, the frequency of Th17 cells was significantly decreased in the intestinal sites in both acarbose and miglitol when treatment started prior to arthritis induction (**Figure 3**). To examine the anti-inflammatory response in the mice, we chose two well-characterized markers, Helios^+^ and CCR6^+^ of Treg cells which have been previously demonstrated to provide robust results and reproducible result in Treg cells. Mice treated with acarbose prior to arthritis induction had significantly increased CD4^+^CD25^+^Foxp3^+^ Treg cells as well as elevation of Helios and CCR6 as demonstrated in both percentage and absolute Treg cell numbers (**Figure 3** and **Table 2**). Treatment with miglitol also observed increased percentage of CD4^+^CD25^+^Foxp3^+^ Tregs (*P <*0.01) and CCR6^+^ (*P <* 0.05) in the mucosal sites (**Figure 3**), but the increased absolute number of Treg cells did not reach statistical significance (**Table 2**). Whereas, neither CD4^+^CD25^+^Foxp3^+^Helios^+^ nor CCR6^+^ response to α-glucosidase inhibitors was observed in the groups when treatment started at arthritis induction (**Figures 3C, D**). This confirmed α-glucosidase inhibitors have a prophylactic effect on lymphocytes resided in the gut lamina propria. α-glucosidase inhibitors suppressed induction of Th17 cells and with a trend towards an increase of Treg cells. As αglucosidase inhibitors modulated Th17/Treg subsets, we addressed the question of whether α-glucosidase inhibitors could affect the gut microbiota at the site which may further adjust the differentiation of Th cells.

**Table 2:** Acarbose induced increase of Treg cells in lamina propria^1^.

	Spleen						Lamina Propria					
	Treg	p*	CCR6^+^Treg	p	Helios^+^Treg	p	Treg	p	CCR6^+^Treg	p	Helios^+^Treg	p
**Before Induction**												
Water	514 ± 64		171 ± 91		355 ± 111		647 ± 182		519 ± 117		360 ± 97	
Miglitol	525 ± 51	0.67	138 ± 22	0.31	385 ± 30	0.44	883 ± 331	0.08	697 ± 281	0.10	430 ± 135	0.23
Acarbose	498 ± 71	0.62	171 ± 107	1.00	349 ± 77	0.20	1,086 ± 274	0.001	908 ± 236	0.000	570 ± 129	0.001
**At Induction**												
Water	496 ± 94		168 ± 91		387 ± 84		550 ± 123		348 ± 74		400 ± 132	
Miglitol	502 ± 59	0.88	163 ± 54	0.87	396 ± 72	0.81	627 ± 239	0.40	483 ± 207	0.08	483 ± 197	0.33
Acarbose	457 ± 76	0.35	159 ± 70	0.81	355 ± 70	0.42	566 ± 209	0.85	348 ± 133	1.00	423 ± 159	0.76

^1^Absolute Treg cell numbers were derived from % of Foxp3^+^ or Foxp3^+^/CCR6^+^ or Foxp3^+^/Helios^+^ CD4^+^/CD25^+^ T cells out of a total of 50,000 cells acquired on flow cytometry. Pooled data of two experiments with n = 7–10 mice in each group. The values are mean ± SD.

*p values are comparison of miglitol or acarbose treated group with water treated.

### Alteration in Microbial Community and Function by α-Glucosidase Inhibitors

**Figure 4:**
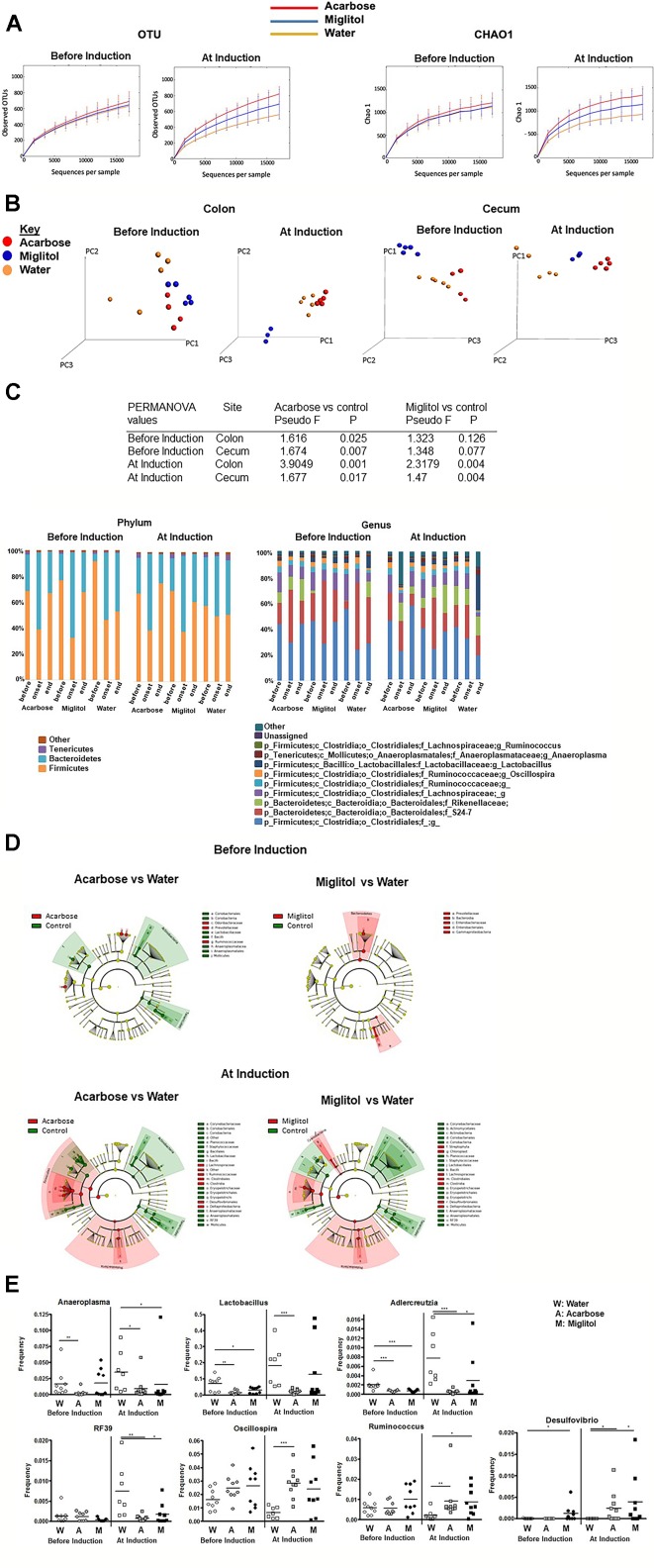
Alteration in microbial community by α-glucosidase inhibitors. Mice were treated with acarbose, miglitol or water as in **Figure 1** (Before Induction or At Induction). **(A)** Alpha diversity of intestinal microbiota as represented by number of observed OTUs and Chao1 estimator. **(B)** Principal coordinate analysis (PCoA) plots displaying Beta diversity of microbiota by treatment group using unweighted UniFrac distances. **(C)** The composition of gut microbiota in colon at the phylum (left) and genus (right) levels. Each phylum/genus is represented by different color and its proportion of relative abundance per sample. Genera were filtered for those with ≥0.1% of total abundance. If full identification was not possible, f_ or g_ alone was used for family or genus, respectively. **(D)** Cladogram representing the most differentially abundant taxa enriched in microbiota from α-glucosidase inhibitors and control mice generated from LEfSe analysis. The central point represents the root of the tree (Bacteria), and each ring represents the next lower taxonomic level (phylum through genus). The diameter of each circle represents the relative abundance of the taxon. **(E)** Relative abundance of *Lactobacillus*, *Anaeroplasma*, *Adlercreutzia, RF39, Oscillospira, Ruminococcus* and *Desulfovibrio*; asterisks indicate level of significance by Mann–Whitney U test. These results consist of the average of two independent experiments, 7–10 mice per group. Mean values ± SEM are plotted; *p < 0.05, **p < 0.01, ***p < 0.001.

Fecal pellets were collected for analysis by V4 rRNA sequencing before treatment (on day −7 or 0), during onset of arthritis (on day 37) and the end of treatment (on day 55). Cecal contents were collected at the end of the experiments (on day 55). Reads were clustered into 1,010  ± 228 OTUs at 97% identity. We then examined whether fecal microbiota richness and diversity, as estimated by the number of observed OTUs and Chao1 estimators, were changed by α-glucosidase inhibitors treatment in different treatment arms (**Figure 4A**). Rarefaction curves plateaued after 15,000 reads per sample, approximating a saturation phase. The variability of OTUs estimated in controls was lower compared to the acarbose groups in animals treated before or at the time of arthritis induction. As shown by the Chao1 estimator, bacterial diversity and richness in CIA control mice with arthritis was significantly lower than in acarbose treatment groups (P < 0.05). All measures indicate a much higher biodiversity within the acarbose treatment than in water control samples (**Figure 4A**).

We next sought to evaluate overall differences in beta-diversity between the microbial communities. We applied the unweighted UniFrac metric (Lozupone and Knight, 2005) to calculate pairwise distances between samples, which takes into account phylogenetic distances among microbes and their presence or absence. As shown from principal coordinate analysis (PCoA) plot (**Figure 4B**), at the end time point a clear separation between the acarbose and the control group was observed in both before and at arthritis induction treatment groups in the colon and cecum (P <0.05, permutation test with pseudo F-ratio). Within the miglitol microbial community cluster, there appears to be clear difference in treatment groups (P < 0.05), but not observed in groups treated before arthritis induction compared with control. Notably in the cecum, we also found significant differences between groups in both before and at arthritis induction treatment groups (P <0.05) (**Figure 4B**). This evidence suggested that the overall composition of the microbial community was significantly changed in the α-glucosidase inhibitor treatment groups.

We hypothesized that there would be compositional changes in the intestinal microbiota between treatment groups. At the phylum level, *Firmicutes* and *Bacteroidetes* were the two largest phyla represented in each group (**Figure 4C**). In the prophylactic groups, *Firmicutes* and *Bacteroidetes* exhibited large shifts during treatment. *Firmicutes* were significantly reduced after arthritis onset and the ratio of *Firmicutes* increased in the α-glucosidase inhibitors treatment groups. In the group of acarbose treatment before arthritis induction, *Firmicutes* was higher before immunization (68.63%), lower at arthritis onset (38.97%) and restored after treatment (66.75%). The microbiota composition was similar in the miglitol treatment group. The proportional abundance of *Firmicutes* was higher before immunization (76.82%) and decreased with arthritis onset (32.48%) and increased after treatment (67.60%). In contrast, *Firmicutes* dropped after immunization but was unable to be restored (52.71%) in controls. This trend was also detected in the treatment groups, at the end of the study α-glucosidase inhibitors restored *Firmicutes* which was significant (P <0.01).

To determine whether the intestinal microbiota contributes directly to arthritis progression, we generated a cladogram with LEfSe to detect bacterial taxa with significantly different abundances between groups (Segata et al., 2011). A comparison between the α-glucosidase inhibitor treatment and control groups showed that 42 OTUs were significantly different from acarbose group and 38 OTUs were in miglitol groups among samples of treatment arms at genus level (**Figures 4C, D**). In addition, in the groups which treatment started prior to arthritis induction, LEfSe identified 19 discriminative features from acarbose groups but 8 OTUs were significantly higher in the miglitol group. As shown above, to identify the effect of α-glucosidase inhibitors on gut bacteria, we found that genus *Lactobacillus, Anaeroplasma, Adlercreutzia*, and *RF39* was significantly higher in control groups than the α-glucosidase inhibitors treated littermates, while *Oscillospira*, *Desulfovibrio* and *Ruminococcus* were enriched in the α-glucosidase inhibitors treated littermates compared with the control groups in the treatment arm (**Figure 4E**). We did not notice any significant difference between acarbose when compared with miglitol. Thus, we identified microbes which were altered between α-glucosidase inhibitors groups and control in the development of arthritis, suggesting roles of α-glucosidase inhibitors in host metabolic development.

## Discussion

In this study, we have shown that administration of α-glucosidase inhibitors, especially acarbose before arthritis induction has a potent modulatory effect upon reversing joint inflammation both clinically and histologically in CIA, but the effect was much less significant when treatment started at the time of arthritis induction. These results are consistent with those by Chen and colleagues (Chen et al., 2015) in which acarbose gained both clinical and histological improvement of CIA. The notable immunological feature in CIA mice is the excessive production of autoantibodies against CII which may initiate arthritis. Our data suggest that high titers of anti-chicken CII antibodies like IgG, IgG1 and IgG2b production in control mice may contribute to the more severe disease. Furthermore, acarbose treated mice produced significantly lower levels of these antibodies, which may prevent the development of arthritis. Another critical feature of CIA is the over expression of pro-inflammatory cytokines that could contribute to inflammation, articular destruction, and the comorbidities associated with arthritis. There is evidence suggesting that IL-9 production in Th17 cells has been related to autoimmune disease including type 1 diabetes (Nowak and Noelle, 2010), asthma (Kearley et al., 2011), and experimental autoimmune encephalomyelitis (Li et al., 2010). Since IL-9 is a Th17-associated cytokine, a few studies evaluated the function of IL-9 in the pathogenesis of RA (Dantas et al., 2015). IL-9 has recently been found to be overexpressed in RA synovial tissues and correlated with the degree of histological organization of B and T cells in ectopic lymphoid structures (Ciccia et al., 2015). Also, IL-9 was related to the presence of RA-related autoantibodies (Hughes-Austin et al., 2013). Our findings suggest that IL-9 is overexpressed in CIA control mice compared to that in mice prophylactic treatment with acarbose. In addition, mice treated with miglitol resulted in significant decreased expression of IL-9, indicating α-glucosidase inhibitors regulating IL-9 development of CIA. Numerous studies suggest that IL-6 contributes to the pathophysiology of RA. An IL-6 inhibitor is a desirable therapeutic option in the treatment of RA (Emery et al., 2008). Both acarbose and miglitol have been reported to suppress IL-6 levels in obese subjects (Arakawa et al., 2008; Derosa et al., 2011). In our study, the concentration of IL-6 was significantly lower in acarbose treated animals, which is consistent with the previous report in CIA mice (Chen et al., 2015).

The imbalance between Th17 and Treg cells has been tightly related to the pathogenesis of RA (McInnes and Schett, 2011) and CIA (Chu et al., 2003; Chu et al., 2007; Annunziato et al., 2009; van den Berg and Miossec, 2009; Morita et al., 2011). In our study, we did not notice any differences of Th17/Treg cells in the spleen between groups regardless whether mice were treated before or at the time of arthritis induction. Notably, our data on α-glucosidase inhibitors suggest that most of its anti-inflammation effect is due to inhibition of intestinal mucosal immunity. α-glucosidase inhibitors suppressed Th17 responses associated with a relative induction of a Treg subset response. The proportion of Th17 cells in the gut LP was significantly lower in the mice treated with acarbose and migltiol before arthritis induction than those treated with drinking water. This suggests that the consumption α-glucosidase inhibitors are not simply immunosuppressive but immunoregulatory because it also favored the anti-inflammatory effect of Treg cells. Recently, it has been demonstrated that Helios-enriched Treg might exert increased suppressive effects (Zabransky et al., 2012; Baine et al., 2013). In addition, Helios expression in CD4^+^ T cells correlated inversely with the serum C-reactive protein level in RA patients (Takatori et al., 2015). CCR6^+^Treg cells preferentially migrated to the colon during inflammation (Kitamura et al., 2010). Prophylactic administration of acarbose not only increased CD4^+^CD25^+^Foxp3^+^ T cells but also elevated Helios and CCR6 in the intestine. Prophylactic administration of miglitol also led to an increased number of CD4^+^CD25^+^Foxp3^+^ T cells and elevated expression of CCR6.

Host-commensal interactions have increasingly been related to the induction of numerous autoimmune inflammatory diseases both in experimental animals and in humans including inflammatory bowel disease (Xavier and Podolsky, 2007), diabetes (Cani et al., 2008), multiple sclerosis (Jangi et al., 2016) and RA (Scher and Abramson, 2011). Previous work has emphasized the anti-inflammatory effect of short chain fatty acids (SCFA) in elevating chronic inflammatory disease, α-glucosidase inhibitors prevent the digestion of the carbohydrates and non-digestible carbohydrates generally provide the main sources of energy for the microbial organism that produce SCFA in the large intestine. Intake of acarbose and miglitol results in larger proportions of undigested carbohydrates reaching the ileum and colon ascendens where they are digested by bacteria to form SCFA (Hanefeld and Schaper, 2008). It has been shown by several studies that SCFA are potent inducer of Treg cells and modulate cytokine production (Arpaia et al., 2013; Furusawa et al., 2013; Harrison et al., 2014; Kim et al., 2014). Considering acarbose is almost not absorbed from the gastrointestinal tract, whereas, miglitol is well absorbed with 100% systemic bioavailability, these two drugs may reestablish the Th17/Treg to a “normal” state in the gut *via* different mechanisms. Smith and colleagues observed that the commensal bacterial metabolites in the colon - SCFA, selectively expand the frequency and number of Treg cells in the large intestine mediated by the G-protein-coupled free fatty acid receptor 43 (GPR43) (Harrison et al., 2014). SCFA, particularly butyrate has been recognized to regulate inflammation and immune responses which up regulated the production of anti-inflammatory cytokines and reduce IL-6 levels, resulting in the induction of CD4^+^CD25^+^ Treg cells (Asarat et al., 2016). Scheppach and colleagues (Scheppach et al., 1988) reported an increase in both butyrate concentration and excretion with the treatment of acarbose. The non-absorbed acarbose reaches colon to provide extra carbohydrates for commensal bacteria to digest for more SCFA production. This may explain the more potent anti-inflammation effects of acarbose than miglitol.

We undertook studies to define the community structure of the fecal microbiome in CIA arthritic models using high-throughput 16S rRNA gene sequencing. We observed more richness within α-glucosidase inhibitors treated groups than the control littermates. At the phylum level, more specific alteration was observed, including decreases in the ratio of member of the *Firmicutes* to *Bacteroidetes* at arthritis onset which was restored after treatment while controls were unable to restore *Firmicutes.* Several intestinal species of *Firmicutes* produce SCFA butyrate, which is known to play a key role in providing major energy source for colonic intestinal epithelial cells maintaining integrity of the gut epithelium, inhibiting inflammatory response, helping regulating the production and development of Treg cells in the colon (Segain et al., 2000).

At genera level, five genera flourished after treatment with α-glucosidase inhibitor, including *Oscillospira*, *Ruminococcus* and *Desulfovibrio*, while the abundance of *Lactobacillus*, *Anaeroplasma*, *Adlercreutzia Ruminococcus* and *RF39* were increased in mice with arthritis. Several intestinal organisms identified in this study as being altered in animal models have been demonstrated to be associated with RA. *Lactobacillus* was over-represented in the fecal, dental, and salivary microbiota of patients with RA than the healthy controls, and its abundance was particularly pronounced in severe cases (Arpaia et al., 2013; Su et al., 2015; Breban et al., 2017). Also, *Lactobacillus* was significantly more abundant in CIA-susceptible mice prior to arthritis onset than in CIA-resistant mice (Jangi et al., 2016), although previous clinical study demonstrated enhanced abundance of *Lactobacillus* with acarbose treatment in prediabetic patients (Zhang et al., 2017). On the other hand, we observed the relative abundance of *Oscillospira* to be greatly enriched following α-glucosidase inhibitors. *Oscillospira* is an enigmatic bacterial genus that has never been cultured, but is recently detected by 16S rRNA gene sequencing. Some *Oscillospira* species are likely to be able to utilize host glycans and can probably produce the SCFA, butyrate (Gophna et al., 2017). *Oscillospira* has been reported to have negative association with inflammatory diseases and lower body mass index, and is positively associated with lean and healthy subjects (Tims et al., 2013; Escobar et al., 2014; Goodrich et al., 2014). Notably, *Oscillospira* is significantly reduced in patients with Crohn’s disease (Walters et al., 2014) and also reduced in pediatric nonalcoholic steatohepatitis, an inflammatory disease of the liver characterized by deposition of large amounts of fat in the liver (Gophna et al., 2017). These studies imply that *Oscillospira* helps in reducing arthritis. In addition, *Ruminococcus* have been reported to be capable of fermenting glucose, xylose and indigestible diet fiber and produce acetate *via* acetyl-CoA from pyruvate and Wood–Ljungdahl pathway (Crost et al., 2013). *Ruminococcus bromii* is suggested as a keystone species for the degradation of resistant starch in the human colon which fermented by intestinal microbiota to secrete SCFA. Scher and colleagues (Scher, 2016) showed a significant lower level in members of the bacterial genus *Akkermansia, Ruminococcus*, and *Pseudobutyrivibrio* in fecal samples from patients with psoriatic arthritis. Also, we found the abundance of genus *Desulfovibrio*, a gram-negative, aerotolerant, non-spore former was at a higher level following α-glucosidase inhibitors than their counterparts. *Desulfovibrio* species work synergistically with *Prevotella* species to degrade mucin (Arumugam et al., 2011). In addition, *Desulfovibrio*, *Prevotella*, *and Oscillibacter* can utilize microbial exopolysaccharides synthesized by *Bifidobacterium* to produce SCFA in the human intestine (Salazar et al., 2008).

Our study has limitations. Our results showed that dysbiosis of gut microflora composition during the course of CIA and the homeostasis was restored by acarbose treatment. These changes are associated with decreased Th17 cells and increased in Treg cells in the gut. However, our study did not provide direct evidence as to which bacterium directly contribute to the inflammation or anti-inflammatory effect, or contribute to maintain the balance between Th17 and Treg cells. Rather, our study shows an association between the reduced inflammation and restored gut microflora in acarbose treated mice. Further studies may need to focus on influence of acarbose on changes of commensal bacteria and their metabolites since commensal bacteria have been shown to selectively expand Treg cells (Smith et al., 2013).

In summary, changes in the gut microbiome and fermentation products in mice treated with acarbose have been associated with reduction of inflammation and with enhanced longevity (Smith et al., 2019) which may be due to decreased inflammation. The anti-arthritis effect of acarbose is best executed in prophylaxis of arthritis in the CIA model but less potent when treatment started at the time of arthritis induction. Considering the clinical usage of acarbose in management of RA, acarbose may have a place in preventing the development of RA in at-risk populations and in maintaining remission status of RA patients who have reached remission after treatment with DMARDs.

## Data Availability Statement

The raw datasets generated for this study can be found in the Bioproject with accession number: PRJNA589659.

## Ethics Statement

The animal study was reviewed and approved by Institutional Animal Care and Use Committee of VA Portland Health Care System (protocol #: 3765-15).

## Author Contributions

LZ, YL, MA and C-QC conceived the project. LZ, PS, XZ, CM, MS, JZ and QX performed the experiments. LZ, LK, EL, MA and C-QC analyzed results. LZ, YL, MA and C-QC wrote the first draft. All authors revised the manuscript.

## Funding

This project was funded by Portland VA Research Foundation and Arthritis and Rheumatic Disease Research Foundation of Oregon Health & Science University.

## Conflict of Interest

The authors declare that the research was conducted in the absence of any commercial or financial relationships that could be construed as a potential conflict of interest.

## References

[B1] Annunziato F. Cosmi L. Liotta F. Maggi E. Romagnani S. (2009). Type 17 T helper cells-origins, features and possible roles in rheumatic disease. Nat. Rev. Rheumatol. 5, 325–331. 10.1038/nrrheum.2009.80 19434074

[B2] Arakawa M. Ebato C. Mita T. Fujitani Y. Shimizu T. Watada H. (2008). Miglitol suppresses the postprandial increase in interleukin 6 and enhances active glucagon-like peptide 1 secretion in viscerally obese subjects. Metabolism 57, 1299–1306. 10.1016/j.metabol.2008.04.027 18702958

[B3] Arpaia N. Campbell C. Fan X. Dikiy S. van der Veeken J. deRoos P. (2013). Metabolites produced by commensal bacteria promote peripheral regulatory T-cell generation. Nature 504, 451–455. 10.1038/nature12726 24226773 PMC3869884

[B4] Arumugam M. Raes J. Pelletier E. Le Paslier D. Yamada T. Mende D. R. (2011). Enterotypes of the human gut microbiome. Nature 473, 174–180. 10.1038/nature09944 21508958 PMC3728647

[B5] Asarat M. Apostolopoulos V. Vasiljevic T. Donkor O. (2016). Short-chain fatty acids regulate cytokines and Th17/Treg cells in human peripheral blood mononuclear cells *in vitro* . Immunol. Invest. 45, 205–222. 10.3109/08820139.2015.1122613 27018846

[B6] Baine I. Basu S. Ames R. Sellers R. S. Macian F. (2013). Helios induces epigenetic silencing of IL2 gene expression in regulatory T cells. J. Immunol. 190, 1008–1016. 10.4049/jimmunol.1200792 23275607 PMC3558938

[B7] Benjamini Y. Hochberg Y. (1995). Controlling the false discovery rate: A practical and powerful approach to multiple testing. J. R. Stat. Soc. Ser. B (Methodological) 57, 289–300. 10.1111/j.2517-6161.1995.tb02031.x

[B8] Bischoff H. (1994). Pharmacology of alpha-glucosidase inhibition. Eur. J. Clin. Invest. 24 Suppl 3, 3–10. 10.1111/j.1365-2362.1994.tb02418.x 8001624

[B9] Breban M. Tap J. Leboime A. Said-Nahal R. Langella P. Chiocchia G. (2017). Faecal microbiota study reveals specific dysbiosis in spondyloarthritis. Ann. Rheum Dis. 76, 1614–1622. 10.1136/annrheumdis-2016-211064 28606969

[B10] Cani P. D. Bibiloni R. Knauf C. Waget A. Neyrinck A. M. Delzenne N. M. (2008). Changes in gut microbiota control metabolic endotoxemia-induced inflammation in high-fat diet-induced obesity and diabetes in mice. Diabetes 57, 1470–1481. 10.2337/db07-1403 18305141

[B11] Caporaso J. G. Kuczynski J. Stombaugh J. Bittinger K. Bushman F. D. Costello E. K. (2010). QIIME allows analysis of high-throughput community sequencing data. Nat. Methods 7, 335–336. 10.1038/nmeth.f.303 20383131 PMC3156573

[B12] Caporaso J. G. Lauber C. L. Walters W. A. Berg-Lyons D. Huntley J. Fierer N. (2012). Ultra-high-throughput microbial community analysis on the Illumina HiSeq and MiSeq platforms. ISME J. 6, 1621–1624. 10.1038/ismej.2012.8 22402401 PMC3400413

[B13] Chen H. H. Chao Y. H. Chen D. Y. Yang D. H. Chung T. W. Li Y. R. (2016). Oral administration of acarbose ameliorates imiquimod-induced psoriasis-like dermatitis in a mouse model. Int. Immunopharmacol. 33, 70–82. 10.1016/j.intimp.2016.02.001 26874324

[B14] Chen H. H. Chen D. Y. Chao Y. H. Chen Y. M. Wu C. L. Lai K. L. (2015). Acarbose decreases the rheumatoid arthritis risk of diabetic patients and attenuates the incidence and severity of collagen-induced arthritis in mice. Sci. Rep. 5, 18288. 10.1038/srep18288 26678745 PMC4683371

[B15] Chu C. Q. Song Z. Mayton L. Wu B. Wooley P. H. (2003). IFNgamma deficient C57BL/6 (H-2b) mice develop collagen induced arthritis with predominant usage of T cell receptor Vbeta6 and Vbeta8 in arthritic joints. Ann. Rheum. Dis. 62, 983–990. 10.1136/ard.62.10.983 12972478 PMC1754310

[B16] Chu C. Q. Swart D. Alcorn D. Tocker J. Elkon K. B. (2007). Interferon-gamma regulates susceptibility to collagen-induced arthritis through suppression of interleukin-17. Arthritis Rheum. 56, 1145–1151. 10.1002/art.22453 17393396

[B17] Ciccia F. Guggino G. Rizzo A. Manzo A. Vitolo B. La Manna M. P. (2015). Potential involvement of IL-9 and Th9 cells in the pathogenesis of rheumatoid arthritis. Rheumatol. (Oxford) 54, 2264–2272. 10.1093/rheumatology/kev252 26178600

[B18] Crost E. H. Tailford L. E. Le Gall G. Fons M. Henrissat B. Juge N. (2013). Utilisation of mucin glycans by the human gut symbiont Ruminococcus gnavus is strain-dependent. PloS One 8, e76341. 10.1371/journal.pone.0076341 24204617 PMC3808388

[B19] Dantas A. T. Marques C. D. da Rocha Junior L. F. Cavalcanti M. B. Goncalves S. M. Cardoso P. R. (2015). Increased Serum Interleukin-9 levels in rheumatoid arthritis and systemic lupus erythematosus: pathogenic role or just an epiphenomenon? Dis. Markers 2015, 519638. 10.1155/2015/519638 26078482 PMC4452366

[B20] Dash R. P. Babu R. J. Srinivas N. R. (2018). Reappraisal and perspectives of clinical drug-drug interaction potential of alpha-glucosidase inhibitors such as acarbose, voglibose and miglitol in the treatment of type 2 diabetes mellitus. Xenobiotica 48, 89–108. 10.1080/00498254.2016.1275063 28010166

[B21] Derosa G. Maffioli P. Ferrari I. Fogari E. D’Angelo A. Palumbo I. (2011). Acarbose actions on insulin resistance and inflammatory parameters during an oral fat load. Eur. J. Pharmacol. 651, 240–250. 10.1016/j.ejphar.2010.11.015 21118681

[B22] DeSantis T. Z. Hugenholtz P. Larsen N. Rojas M. Brodie E. L. Keller K. (2006). Greengenes, a chimera-checked 16S rRNA gene database and workbench compatible with ARB. Appl. Environ. Microbiol. 72, 5069–5072. 10.1128/AEM.03006-05 16820507 PMC1489311

[B23] Dixon P. (2003). VEGAN, a package of R functions for community ecology. J. Veg. Sci. 14, 927–930. 10.1111/j.1654-1103.2003.tb02228.x

[B24] Emery P. Keystone E. Tony H. P. Cantagrel A. van Vollenhoven R. Sanchez A. (2008). IL-6 receptor inhibition with tocilizumab improves treatment outcomes in patients with rheumatoid arthritis refractory to anti-tumour necrosis factor biologicals: results from a 24-week multicentre randomised placebo-controlled trial. Ann. Rheum. Dis. 67, 1516–1523. 10.1136/ard.2008.092932 18625622 PMC3811149

[B25] Escobar J. S. Klotz B. Valdes B. E. Agudelo G. M. (2014). The gut microbiota of Colombians differs from that of Americans, Europeans and Asians. BMC Microbiol. 14, 311. 10.1186/s12866-014-0311-6 25495462 PMC4275940

[B26] Furusawa Y. Obata Y. Fukuda S. Endo T. A. Nakato G. Takahashi D. (2013). Commensal microbe-derived butyrate induces the differentiation of colonic regulatory T cells. Nature 504, 446–450. 10.1038/nature12721 24226770

[B27] Goodrich J. K. Waters J. L. Poole A. C. Sutter J. L. Koren O. Blekhman R. (2014). Human genetics shape the gut microbiome. Cell 159, 789–799. 10.1016/j.cell.2014.09.053 25417156 PMC4255478

[B28] Gophna U. Konikoff T. Nielsen H. B. (2017). Oscillospira and related bacteria - From metagenomic species to metabolic features. Environ. Microbiol. 19, 835–841. 10.1111/1462-2920.13658 28028921

[B29] Hanefeld M. Schaper F. (2008). Acarbose: oral anti-diabetes drug with additional cardiovascular benefits. Expert Rev. Cardiovasc. Ther. 6, 153–163. 10.1586/14779072.6.2.153 18248270

[B30] Harrison D. E. Strong R. Alavez S. Astle C. M. DiGiovanni J. Fernandez E. (2019). Acarbose improves health and lifespan in aging HET3 mice. Aging Cell 18, e12898. 10.1111/acel.12898 30688027 PMC6413665

[B31] Harrison D. E. Strong R. Allison D. B. Ames B. N. Astle C. M. Atamna H. (2014). Acarbose, 17-alpha-estradiol, and nordihydroguaiaretic acid extend mouse lifespan preferentially in males. Aging Cell 13, 273–282. 10.1111/acel.12170 24245565 PMC3954939

[B32] Hughes-Austin J. M. Deane K. D. Derber L. A. Kolfenbach J. R. Zerbe G. O. Sokolove J. (2013). Multiple cytokines and chemokines are associated with rheumatoid arthritis-related autoimmunity in first-degree relatives without rheumatoid arthritis: Studies of the Aetiology of Rheumatoid Arthritis (SERA). Ann. Rheum. Dis. 72, 901–907. 10.1136/annrheumdis-2012-201505 22915618 PMC3726193

[B33] Jangi S. Gandhi R. Cox L. M. Li N. von Glehn F. Yan R. (2016). Alterations of the human gut microbiome in multiple sclerosis. Nat. Commun. 7, 12015. 10.1038/ncomms12015 27352007 PMC4931233

[B34] Joshi S. R. Standl E. Tong N. Shah P. Kalra S. Rathod R. (2015). Therapeutic potential of alpha-glucosidase inhibitors in type 2 diabetes mellitus: an evidence-based review. Expert Opin. Pharmacother. 16, 1959–1981. 10.1517/14656566.2015.1070827 26255950

[B35] Kearley J. Erjefalt J. S. Andersson C. Benjamin E. Jones C. P. Robichaud A. (2011). IL-9 governs allergen-induced mast cell numbers in the lung and chronic remodeling of the airways. Am. J. Respir. Crit. Care Med. 183, 865–875. 10.1164/rccm.200909-1462OC 20971830 PMC3385369

[B36] Kim C. H. Park J. Kim M. (2014). Gut microbiota-derived short-chain Fatty acids, T cells, and inflammation. Immune Netw. 14, 277–288. 10.4110/in.2014.14.6.277 25550694 PMC4275385

[B37] Kitamura K. Farber J. M. Kelsall B. L. (2010). CCR6 marks regulatory T cells as a colon-tropic, IL-10-producing phenotype. J. Immunol. 185, 3295–3304. 10.4049/jimmunol.1001156 20720211 PMC3932491

[B38] Li H. Nourbakhsh B. Ciric B. Zhang G. X. Rostami A. (2010). Neutralization of IL-9 ameliorates experimental autoimmune encephalomyelitis by decreasing the effector T cell population. J. Immunol. 185, 4095–4100. 10.4049/jimmunol.1000986 20805418 PMC2978501

[B39] Lozupone C. Knight R. (2005). UniFrac: a new phylogenetic method for comparing microbial communities. Appl. Environ. Microbiol. 71, 8228–8235. 10.1128/AEM.71.12.8228-8235.2005 16332807 PMC1317376

[B40] McInnes I. B. Schett G. (2011). The pathogenesis of rheumatoid arthritis. N. Engl. J. Med. 365, 2205–2219. 10.1056/NEJMra1004965 22150039

[B41] Morita Y. Ismail D. M. Elkon K. B. Chu C. Q. (2011). Dichotomous response to transforming growth factor beta after T cell receptor activation by naive CD4+ T cells from DBA/1 mice: enhanced retinoic acid receptor-related orphan nuclear receptor gammat expression yet reduced FoxP3 expression. Arthritis Rheum. 63, 118–126. 10.1002/art.27759 20862680 PMC3107673

[B42] Nowak E. C. Noelle R. J. (2010). Interleukin-9 as a T helper type 17 cytokine. Immunology 131, 169–173. 10.1111/j.1365-2567.2010.03332.x 20673237 PMC2967262

[B43] Phillips R. (2015). Rheumatoid arthritis: microbiome reflects status of RA and response to therapy. Nat. Rev. Rheumatol. 11, 502. 10.1038/nrrheum.2015.109 26241185

[B44] Rogers G. B. (2015). Germs and joints: the contribution of the human microbiome to rheumatoid arthritis. Nat. Med. 21, 839–841. 10.1038/nm.3916 26248263

[B45] Salazar N. Gueimonde M. Hernandez-Barranco A. M. Ruas-Madiedo P. de los Reyes-Gavilan C. G. (2008). Exopolysaccharides produced by intestinal Bifidobacterium strains act as fermentable substrates for human intestinal bacteria. Appl. Environ. Microbiol. 74, 4737–4745. 10.1128/AEM.00325-08 18539803 PMC2519331

[B46] Scheppach W. Fabian C. Sachs M. Kasper H. (1988). The effect of starch malabsorption on fecal short-chain fatty acid excretion in man. Scand. J. Gastroenterol. 23, 755–759. 10.3109/00365528809093945 3175536

[B47] Scher J. U. Abramson S. B. (2011). The microbiome and rheumatoid arthritis. Nat. Rev. Rheumatol. 7, 569–578. 10.1038/nrrheum.2011.121 21862983 PMC3275101

[B48] Scher J. U. (2016). The microbiome in celiac disease: beyond diet-genetic interactions. Cleve Clin. J. Med. 83, 228–230. 10.3949/ccjm.83a.15123 26974994

[B49] Segain J. P. Raingeard de la Bletiere,. D. Bourreille A. Leray V. Gervois N. Rosales C. (2000). Butyrate inhibits inflammatory responses through NFkappaB inhibition: implications for Crohn’s disease. Gut 47, 397–403. 10.1136/gut.47.3.397 10940278 PMC1728045

[B50] Segata N. Izard J. Waldron L. Gevers D. Miropolsky L. Garrett W. S. (2011). Metagenomic biomarker discovery and explanation. Genome Biol. 12, R60. 10.1186/gb-2011-12-6-r60 21702898 PMC3218848

[B51] Sels J.-P.J.E. Huijberts M. S. P. Wolffenbuttel B. H. R. (1999). Miglitol, a new a-glucosidase inhibitor. Expert Opin. Pharmacother. 1, 149–156. 10.1517/14656566.1.1.149 11249557

[B52] Smith P. M. Howitt M. R. Panikov N. Michaud M. Gallini C. A. Bohlooly Y. M. (2013). The microbial metabolites, short-chain fatty acids, regulate colonic Treg cell homeostasis. Science 341, 569–573. 10.1126/science.1241165 23828891 PMC3807819

[B53] Smith B. J. Miller R. A. Ericsson A. C. Harrison D. C. Strong R. Schmidt T. M. (2019). Changes in the gut microbiome and fermentation products concurrent with enhanced longevity in acarbose-treated mice. BMC Microbiol. 19, 130. 10.1186/s12866-019-1494-7 31195972 PMC6567620

[B54] Su B. Liu H. Li J. Sunli Y. Liu B. Liu D. (2015). Acarbose treatment affects the serum levels of inflammatory cytokines and the gut content of bifidobacteria in Chinese patients with type 2 diabetes mellitus. J. Diabetes 7, 729–739. 10.1111/1753-0407.12232 25327485

[B55] Takatori H. Kawashima H. Matsuki A. Meguro K. Tanaka S. Iwamoto T. (2015). Helios enhances treg cell function in cooperation with FoxP3. Arthritis Rheumatol. 67, 1491–1502. 10.1002/art.39091 25733061

[B56] Tims S. Derom C. Jonkers D. M. Vlietinck R. Saris W. H. Kleerebezem M. (2013). Microbiota conservation and BMI signatures in adult monozygotic twins. ISME J. 7, 707–717. 10.1038/ismej.2012.146 23190729 PMC3603393

[B57] van den Berg W. B. Miossec P. (2009). IL-17 as a future therapeutic target for rheumatoid arthritis. Nat. Rev. Rheumatol. 5, 549–553. 10.1038/nrrheum.2009.179 19798029

[B58] Walters W. A. Xu Z. Knight R. (2014). Meta-analyses of human gut microbes associated with obesity and IBD. FEBS Lett. 588, 4223–4233. 10.1016/j.febslet.2014.09.039 25307765 PMC5050012

[B59] Williams R. O. Feldmann M. Maini R. N. (1992). Anti-tumor necrosis factor ameliorates joint disease in murine collagen-induced arthritis. Proc. Natl. Acad. Sci. U. S. A. 89, 9784–9788. 10.1073/pnas.89.20.9784 1409699 PMC50217

[B60] Xavier R. J. Podolsky D. K. (2007). Unravelling the pathogenesis of inflammatory bowel disease. Nature 448, 427–434. 10.1038/nature06005 17653185

[B61] Zabransky D. J. Nirschl C. J. Durham N. M. Park B. V. Ceccato C. M. Bruno T. C. (2012). Phenotypic and functional properties of Helios+ regulatory T cells. PloS One 7, e34547. 10.1371/journal.pone.0034547 22479644 PMC3316700

[B62] Zhang X. Zhang D. Jia H. Feng Q. Wang D. Liang D. (2015). The oral and gut microbiomes are perturbed in rheumatoid arthritis and partly normalized after treatment. Nat. Med. 21, 895–905. 10.1038/nm.3914 26214836

[B63] Zhang X. Fang Z. Zhang C. Xia H. Jie Z. Han X. (2017). Effects of acarbose on the gut microbiota of prediabetic patients: a randomized, double-blind, controlled crossover trial. Diabetes Ther. 8, 293–307. 10.1007/s13300-017-0226-y 28130771 PMC5380489

